# Sensory and decisional components of endogenous attention are dissociable

**DOI:** 10.1152/jn.00257.2019

**Published:** 2019-07-03

**Authors:** Sanjna Banerjee, Shrey Grover, Suhas Ganesh, Devarajan Sridharan

**Affiliations:** Centre for Neuroscience, Indian Institute of Science, Bangalore, India

**Keywords:** attention mechanisms, criteria, multialternative decisions, psychophysical models, signal detection theory, top-down attention

## Abstract

Endogenous cueing of attention enhances sensory processing of the attended stimulus (perceptual sensitivity) and prioritizes information from the attended location for guiding behavioral decisions (spatial choice bias). Here, we test whether sensitivity and bias effects of endogenous spatial attention are under the control of common or distinct mechanisms. Human observers performed a multialternative visuospatial attention task with probabilistic spatial cues. Observers’ behavioral choices were analyzed with a recently developed multidimensional signal detection model (the m-ADC model). The model effectively decoupled the effects of spatial cueing on sensitivity from those on spatial bias and revealed striking dissociations between them. Sensitivity was highest at the cued location and not significantly different among uncued locations, suggesting a spotlight-like allocation of sensory resources at the cued location. On the other hand, bias varied systematically with cue validity, suggesting a graded allocation of decisional priority across locations. Cueing-induced modulations of sensitivity and bias were uncorrelated within and across subjects. Bias, but not sensitivity, correlated with key metrics of prioritized decision-making, including reaction times and decision optimality indices. In addition, we developed a novel metric, differential risk curvature, for distinguishing bias effects of attention from those of signal expectation. Differential risk curvature correlated selectively with m-ADC model estimates of bias but not with estimates of sensitivity. Our results reveal dissociable effects of endogenous attention on perceptual sensitivity and choice bias in a multialternative choice task and motivate the search for the distinct neural correlates of each.

**NEW & NOTEWORTHY** Attention is often studied as a unitary phenomenon. Yet, attention can both enhance the perception of important stimuli (sensitivity) and prioritize such stimuli for decision-making (bias). Employing a multialternative spatial attention task with probabilistic cueing, we show that attention affects sensitivity and bias through dissociable mechanisms. Specifically, the effects on sensitivity alone match the notion of an attentional “spotlight.” Our behavioral model enables quantifying component processes of attention, and identifying their respective neural correlates.

## INTRODUCTION

Attention is the remarkable cognitive capacity that enables us to select and process relevant information in our sensory environment. In laboratory tasks, endogenous or voluntary attention is typically engaged by cues that are predictive of upcoming stimuli or events of interest. Numerous studies have explored the behavioral and neural correlates of endogenous cueing of attention and have reported the systematic effects of cueing both on sensory processing (Bashinski and Bacharach 1980; Cohen and Maunsell 2009; Hawkins et al. 1990; Kastner and Ungerleider 2000; Luck et al. 1996; McAdams and Maunsell 1999; Palmer and Moore 2009) and decision-making (Baruni et al. 2015; Moran and Desimone 1985; Müller and Findlay 1987; Palmer 1994; Shaw 1984). Signal detection theory (SDT), a highly successful framework for the analysis of behavior (Ashby 1992; Carandini and Churchland 2013; Cohen and Maunsell 2009; Graham 1989; Green and Swets 1966; Hawkins et al. 1990; Macmillan and Creelman 2005; Pestilli et al. 2011; Shaw 1984), provides two key metrics for quantifying sensory and decisional effects of endogenous attention: *1*) perceptual sensitivity (d′), which measures the improvement in sensory processing (signal-to-noise ratio) of the attended stimulus; and *2*) choice bias (or criterion), which measures the priority afforded to attended information for guiding behavioral decisions (Buschman and Kastner 2015; Carandini and Churchland 2013). Whether the effects of endogenous attention on sensitivity and bias are mediated by common or distinct brain regions and mechanisms remains a topic of active research (Buschman and Kastner 2015; Eckstein et al. 2013; Krauzlis et al. 2014; Luck et al. 1996; Luo and Maunsell 2015).

On the one hand, several studies suggest that the same brain regions may mediate sensory and decisional effects of attention. For instance, occipital and temporal areas, typically associated with sensory (visual form) processing, are known to exhibit criterion-related activity (Li et al. 2012; White et al. 2012) in two-alternative forced choice (2-AFC) perceptual discrimination tasks. Similarly, microstimulating the frontal eye field (FEF) has been shown to modulate neuronal firing rates (and signal-to-noise ratio) in V4 neurons, suggesting a key role of the FEF in sensitivity modulation (Moore and Armstrong 2003; Noudoost et al. 2010). Nonetheless, the FEF has also been implicated in modulating decision criteria in a speed discrimination task (Ferrera et al. 2009). Similarly, activity in other frontal areas, including the anterior prefrontal cortex and dorsolateral prefrontal cortex, associated with early representation of visual stimuli is also known to modulate with criterion changes and decision confidence in a range of 2-AFC perceptual decision tasks, involving stimulus detection, contrast, or orientation discrimination (de Lafuente and Romo 2005; Fleming et al. 2010; Katsuki and Constantinidis 2012; Rahnev et al. 2016). Following these reports, a recent study showed that lateral prefrontal cortical activity reflects modulations of both sensitivity and criteria in a 2-AFC attention task involving orientation change detection (Luo and Maunsell 2018). Along the same lines, parietal cortical areas including the intraparietal sulcus and the lateral intraparietal area not only show decision-related activity (Kiani and Shadlen 2009; Platt and Glimcher 1999; Rorie and Newsome 2005; Shadlen and Newsome 2001) but also closely predict visual cortical responses during stimulus anticipation (Bressler et al. 2008). Similarly, recent literature suggests that superior colliculus (SC), a midbrain structure, could be involved in the modulation of both sensitivity and choice criteria (Crapse et al. 2018; Lovejoy and Krauzlis 2017) during discrimination and detection tasks.

On the other hand, other studies have suggested that sensitivity and bias effects of attention are mediated by distinct brain regions. For instance, Luo and Maunsell (2015) suggested that visual cortical (area V4) neurons are specifically modulated by attentional changes in sensitivity but not criteria. Similarly, a recent modeling study (Sridharan et al. 2017) reanalyzed behavioral data from previous perceptual detection and discrimination studies that either microstimulated or inactivated the SC and concluded that the SC is involved selectively in modulating bias, but not sensitivity, in attention tasks. These results were confirmed by other experimental studies, which showed that SC activity during perceptual detection, or discrimination, was correlated with decision criteria alone (Crapse et al. 2018; Grimaldi et al. 2018). Similarly, other recent studies have suggested a specific role for the striatum in modulating decision criteria, rather than sensitivity (Wang et al. 2018).

While there is active interest in identifying the specific neural bases of sensitivity and bias, the diversity of task and reward paradigms employed in the aforementioned studies render these behaviors challenging to analyze and compare in a common, normative framework (Chanes et al. 2013; Luo and Maunsell 2015; see also discussion). Perhaps, as a result, previous studies have reported directly contradictory results, for instance, regarding the involvement of V4 (Baruni et al. 2015; Luo and Maunsell 2015) or the SC (Lovejoy and Krauzlis 2017; Sridharan et al. 2017) in sensitivity versus bias modulation by attention.

**Fig. 1.**
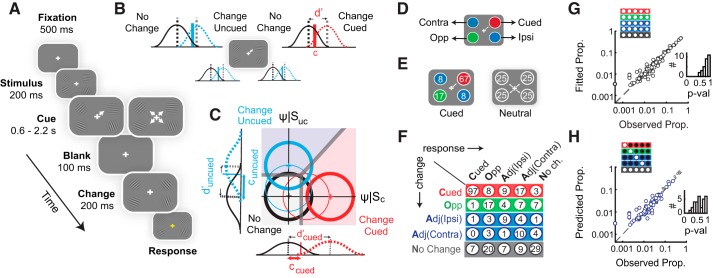
A model for analyzing behavior in multialternative attention tasks. *A*: a probabilistically cued attention task involving change detection. After fixation (500 ms), 4 Gabor patches appeared (200 ms) followed by an attentional cue (central arrow). After a variable delay all patches disappeared briefly (100 ms). Upon reappearance, either 1 of 4 patches had changed in orientation (“change”) or none had changed (“no change”). After 200 ms, the subject had to indicate the location of change, or no change, by pressing 1 of 5 buttons. *B*: multiple 1-dimensional signal detection models representing independent change versus no change decisions at each location. Black Gaussians: noise distribution for “no change” trials. Red and blue Gaussians: signal distribution for “change” trials at the cued location and uncued location, respectively. Dashed vertical lines: means of signal and noise distributions, whose difference quantifies sensitivity (d′) at each location. Solid vertical lines: criterion (detection threshold) at each location (c). *C*: multidimensional (m-ADC) signal detection model for changes at the cued and one uncued location. Orthogonal axes: decision variables at each location. Circles: Gaussian decision variable distribution for “change” trials at the cued location (red), uncued location (light blue), and “no change” trials (black). Thin and thick contours: lower and higher sensitivity values respectively. Thick gray lines: decision manifold delineating cued, uncued and no change decision domains. Gaussians along the axes: marginal densities at each location. *D*: color scheme denoting different locations. Red: cued location (Cued); green: opposite to cue (Opp); blue: adjacent to cue, either in the same (Adj-Ipsi) or contralateral hemifield (Adj-Contra). *E*: proportion of change events at each location for blocks with high cue validity (*left*) and neutrally cued blocks (*right*). *F*: contingency table from a representative behavioral session. Rows: change locations; columns: response locations, both relative to the cue. Color conventions are as in *D*. Gray: no change. *G*: response proportions (gray circles) fitted with the 4-ADC model plotted against actual response proportions. Data points: response probabilities for each stimulus-response contingency at each angle (across *n* = 30 subjects). *Top inset*: subset of contingencies used for fitting. *Right inset*: distribution of goodness of fit *P* values across subjects. *H*: predicted proportions (blue circles) plotted against actual response proportions by fitting hits and false alarms (details in text). Small gray circles: fitted proportions. Other conventions are as in *G*.

Here, we employed an endogenous attention task, with spatial probabilistic cueing (Fig. 1*A*), that involves detecting changes in target orientation at one of several locations. Our task is a variant of the standard Posner cueing task with multiple stimulus locations, which is ubiquitous in attention literature (Cavanaugh and Wurtz 2004; Cohen and Maunsell 2009; Corbetta et al. 2000; Williford and Maunsell 2006). However, in practice, such tasks are challenging to analyze with conventional SDT models because, even in their simplest form, such tasks are multialternative tasks. They require a choice among several (at least 3 or more) alternatives on each trial: event at the cued location, event at one or more uncued location(s) or no event. These tasks cannot be correctly analyzed with a combination of one-dimensional SDT models (Fig. 1*B*) because such a formulation assumes that the decision (event versus no event) at each location is independent of the decisions at other locations; the latter assumption is not true for multialternative tasks (see also Sridharan et al. 2014).

To address this challenge, we analyzed behavioral data in our task with a recently developed multidimensional signal detection model (the m-ADC model), which overcomes the pitfalls of analyses with one-dimensional models (Sridharan et al. 2014, 2017) (Fig. 1*C*). The model decouples, and separately quantifies, sensitivity and bias from participants’ responses in the stimulus-response contingency table (Fig. 1*F*). With this task and model, we asked whether endogenous cueing of attention engages common or distinct mechanisms for modulating sensitivity and bias. In addition, we developed a novel bias measure that decouples the effects of attention from signal expectation, an emerging topic of interest (Simon et al. 2019; Summerfield and Egner 2009). The results reveal dissociable effects of endogenous cueing on sensitivity and bias modulation during predictively cued endogenous attention tasks, in human participants.

## MATERIALS AND METHODS

### Participants

Thirty-seven subjects (21 men; age range: 19–60 yr; median age: 22 yr) with no known history of neurological disorders and with normal or corrected-to-normal vision participated in the experiment. All participants provided written informed consent, and all experimental procedures were approved by the Institute Human Ethics Committee at the Indian Institute of Science, Bangalore. Seven subjects were excluded from analysis because of improper gaze fixation (see *Eye tracking*). Data from 30 subjects were included in the final analysis.

### m-ADC Model Description

SDT provides a rigorous framework for quantifying perceptual sensitivity (e.g., the quality of sensory information processing) as distinct from choice bias (e.g., the relative weighting of sensory evidence from different locations) with behavioral data. Sensitivity is quantified with an index of discriminability (d′) that measures how readily a signal event (here, change) can be distinguished from noise (no change) at each location. Bias is quantified with a choice criterion that represents a threshold for the amount of evidence needed at a particular location before the subject decides that the event of interest (here, the change) occurred at that location. When a stimulus at a particular location is attended, it is expected that either one or both of these components are altered: perceptual sensitivity for the attended target may increase (d′) and/or greater choice bias may be afforded to target-related information. Conventional, one-dimensional SDT models do not suffice to quantify sensitivity and bias in “standard” spatial attention tasks (e.g., Fig. 1*A*): even the simplest version of this task, with only two potential stimulus locations (cued and uncued), is a multialternative task that requires the subject to make one of three choices: change at the cued location, change at the uncued location, or no change. Such tasks cannot be adequately fit with a combination of one-dimensional signal detection models (Fig. 1*B*); the reasons are elaborated in Sridharan et al. (2014) (p. 3–5).

We recently developed a behavioral model based on a multidimensional extension to SDT, the m-ADC model, to quantify sensitivity and bias in standard attention tasks involving any number of stimulus alternatives (Sridharan et al. 2014); such tasks are extensively used in human and nonhuman primate attention studies (Cohen and Maunsell 2009; Corbetta et al. 2000; Lovejoy and Krauzlis 2017; Zénon and Krauzlis 2012). Here, we recapitulate essential features of the model and present an overview of how the model estimates sensitivity and bias from observers’ behavioral responses in such attention tasks; a detailed, quantitative description of the model is available in Sridharan et al. (2014, 2017).

Consider a five-alternative attention task (Fig. 1*A*). In this task, the subject has to detect an orientation change that can occur at any one (or none) of four locations and indicate which of the five possible events occurred. This entails making a decision by comparing perceptual evidence for the change event at each of the four locations as well as the no change event. Five categories of stimulus-response contingencies occur in the 5 × 5 contingency table (Fig. 1*F*): *1*) hits (elements along the diagonal, except the *bottom right*), wherein the subject correctly localizes the change; *2*) correct rejections (the *bottom right* element), wherein the subject correctly reports a no change event; *3*) misses (*last column*, except the *bottom right* element), wherein the subject misses the change event and reports no change; *4*) false alarms (*last row*, except the *bottom right* element) wherein the subject indicates change at a location, when no changes occurred at any location; and *5*) mis-localizations (all others), wherein the subject correctly reports a change event but localizes it to an incorrect location. Fitting the m-ADC model requires computing the proportion of these different categories of responses in the contingency table for each subject. The proportion of hits and false alarms at each location determines the subject’s perceptual sensitivity to changes at that location. Nevertheless, an accurate estimate of sensitivity requires factoring in idiosyncratic, as well as cue-induced, biases that are part of subjects’ decision-making strategies. The proportion of false alarms at each location is indicative (although not entirely informative) of these biases. The m-ADC model defines formal mathematical relationships linking sensitivities and criteria at each location to the proportions of each type of response (Sridharan et al. 2014, p. 5–7).

To provide an intuition for how the m-ADC model relates sensitivities and criteria to response proportions, consider the schematic of a two-dimensional signal-detection model for the 2-ADC task (task with two potential change locations and a no change response; Fig. 1*C*). In this model, the observer’s decision is modeled in a two-dimensional decision space, based on a bivariate decision variable (**Ψ**). The decision variable components for the cued and uncued locations, represented along the *x*- and *y*-axes respectively (Fig. 1*C*), are Gaussian random variables that take on values on each trial according to which event occurred on that trial; we term these components Ψ_cued_ and Ψ_uncued_. Their values are indicative of the strength of the perceived signal at each location. The decision variable distribution for the “no change” event has a mean of zero as is conventional in SDT and is therefore centered at the origin (Fig. 1*C*, black distribution). In case of a change event (signal) at the cued location, the decision variable distribution translates along the *x*-axis (Fig. 1*C*, red distribution; **Ψ**|S_C_), and in case of a change event at the uncued location, the decision variable distribution translates along the *y*-axis (Fig. 1*C*, blue distribution; **Ψ**|S_UC_). The separation of the signal distribution at each location (Fig. 1*C*, red or blue distributions) from the noise distribution (Fig. 1*C*, black distribution) quantifies the subject’s perceptual sensitivity for detecting changes at the respective location (d′_cued_ and d′_uncued_). Geometrically, this corresponds to the distance of the mean of each signal distribution (measured in units of noise standard deviation) from the mean of the noise distribution.

The m-ADC model posits that, to decide where the change occurred, the subject adopts two threshold values, c_cued_ and c_uncued_ (criteria). The subject’s decision is modeled as follows: events that result in Ψ_cued_ exceeding c_cued_ produce a localization response indicating “change” at the cued location, provided Ψ_uncued_ does not exceed c_uncued_. Conversely, events that result in Ψ_uncued_ exceeding c_uncued_ produce a localization response at the uncued location, provided Ψ_cued_ does not exceed c_cued_. If neither Ψ_cued_ nor Ψ_uncued_ exceed criteria, c_cued_ and c_uncued_, respectively, the subject indicates a “no change” response. If both Ψ_cued_ and Ψ_uncued_ exceed criteria c_cued_ and c_uncued_, respectively, then the subject indicates a location of change at which Ψ exceeds the respective c by a larger value (Ψ_cued_ − c_cued_ <> Ψ_uncued_ – c_uncued_). Geometrically, this decision rule corresponds to the observer making a choice among three alternatives, based on a set of planar decision boundaries (Fig. 1*C*, thick solid gray lines) that divide the decision space into three decision zones corresponding to change at the cued location (Fig. 1*C*, red shading), change at the uncued location (Fig. 1*C*, blue shading), and no change (Fig. 1*C*, gray shading). These decision boundaries belong to the family of optimal decision surfaces, are parameterized by the two decision criteria (c_cued_ and c_uncued_), and can be related analytically to sensory evidence, priors, and payoffs at each location (Sridharan et al. 2014, p. 4-6). The integral of the decision variable distribution (**Ψ**|S_C_, **Ψ**|S_UC_), within each decision zone, represents the probability of the corresponding choice (change at cued location, uncued location, or no change).

m-ADC model parameters, perceptual sensitivities (d′_cued_ and d′_uncued_), and criteria (c_cued_ and c_uncued_) at each location, are estimated to fit response probabilities in the 5 × 5 contingency table, with a maximum likelihood (ML) estimation procedure (Sridharan et al. 2014). Bias metrics are computed from estimates of d′ and c using choice criterion and likelihood ratio measures (described in *Computing psychophysical parameters and their modulations*).

In this study, we extend the m-ADC model by deriving, de novo, an optimal decision rule for task designs that employ the method of constant stimuli. In these tasks, performance is measured by presenting stimuli at various, unpredictable strengths (e.g., contrast or magnitude of orientation change) at each location. These include, for example, attention tasks in which the full psychometric function is measured at both cued and uncued locations. The decision rule is constructed from optimal decision theory for minimizing Bayesian risk or maximizing Bayesian utility, and the resultant optimal decision manifold is shown in Supplemental Fig. S1*E*; (all Supplemental Materials are available at https://figshare.com/s/b4b1f34ae4087420bd85). We derive the decision rule in detail in Supplemental Data: Appendix.

### Experimental Design

#### Task.

Subjects were tested on a cued, five-alternative change detection task (Fig. 1*A*). Subjects were seated in an isolated room, with head positioned on a chin rest 60 cm from the center of a contrast calibrated visual display (22-in. LG LCD monitor, X-Rite i1 Spectrophotometer). Stimuli were programmed with Psychtoolbox (version 3.0.11) (Brainard 1997) using MATLAB R2014b (Natick, MA). Responses were recorded with an RB-840 response box (Cedrus). Subjects were instructed to maintain fixation on a central fixation cross during the experiment. Fixation was monitored with a 60-Hz infrared eyetracker (Gazepoint GP3). Subjects began the task by fixating on a fixation cross at the center of a gray screen (0.5° diameter). Following 200 ms, four high-contrast Gabor patches appeared, one in each visual quadrant (Fig. 1*A*) at a distance of 3.5° from the fixation cross (Gabor standard deviation = 0.6°; grating spatial frequency, 2 cycles/°; luminance L_max_ = 42.3 cd/m^2^; L_min_ = 0.1 cd/m^2^; and background luminance: 10.0 cd/m^2^). The orientation of each Gabor patch was drawn from a uniform random distribution (within a range of 0–180°), independently of the other patches, and pseudorandomized across trials. After another 500 ms, a central cue (directed line segment, 0.37° in length) appeared. After a variable delay (600–2,200 ms, drawn from an exponential distribution), the stimuli briefly disappeared (100 ms) and reappeared; stimuli persisted on the screen until the subject’s response. Following reappearance either one of the four stimuli had changed in orientation or none had changed. The subject had to indicate the location of change, or indicate “no change” by pressing one of five buttons on the response box; the locations of the response buttons were congruent with those of the four potential change locations (Supplemental Fig. S1*F*).

All trials were cued toward one of the four locations, but an orientation change occurred only in a subset of trials. We term trials in which a change in orientation occurred in one of the four gratings as “change” trials, and trials in which no change in orientation occurred as “catch” or “no change” trials. Twenty-five percent of all trials were no change trials, and the remaining 75% were change trials. We term the location toward which the cue was directed as the “cued” (C) location, the location diagonally opposite to the cued location as the “opposite” (O) location, and two other locations as “adjacent-ipsilateral” (A-I) or “adjacent-contralateral” (A-C) locations, depending on whether these were in the same or opposite visual hemifield, respectively, to the cued location. Changes occurred at the cued location on two-thirds of the change trials, at the opposite location on one-sixth of the change trials, and at each of the adjacent locations on one-twelfth of the change trials. Thus the cue had a conditional validity of 67% (Fig. 1*E*, *left*) on change trials, and an overall validity of 50%. The experiment was run in 6 blocks of 48 trials each (total 288 trials per subject), with no feedback. In a training session before the experiment, subjects completed 96 trials (2 blocks) with explicit feedback provided at the end of each trial about the location of the change and the correctness of their response. Data from these training blocks were not used for further analyses.

Ten subjects were tested on a version of the task that incorporated neutrally cued blocks. In this task, subjects were tested on a total of eight experimental blocks (48 trials each; total 384 trials), with four blocks comprising predictively cued trials (as before) and the remaining four blocks comprised neutrally cued trials. On neutrally cued trials, the cue comprised of four directed line segments, each pointing toward one of the stimuli in each of the four quadrants (Fig. 1*A*), and changes were equally likely at all four locations (Fig. 1*E*, *right*). Subjects were informed by on-screen instructions before the beginning of each block as to whether it was a predictive cueing or neutral cueing block, and the order of blocks were counterbalanced and pseudorandomized across subjects. All other training and testing protocols remained the same as before.

#### Eye tracking.

Subjects’ gaze was binocularly tracked, and the deviation in their gaze from the fixation cross was recorded and stored in degrees. Trials in which the eye position deviated by more than 2° from fixation either in the *x*- or in the *y*-direction, from the onset of the Gabors until the final response, were removed from further analysis. Blinks were detected during the cue period based on custom MATLAB code using the eyetracker data as input. All of our subjects were South Asian, and several exhibited dark pigmentation of the iris, rendering it challenging to distinguish from the pupil. Hence, the contrast of the pupil (relative to the iris) was weak, and the tracker occasionally lost the location of the pupil; trials in which blinks occurred or the tracker lost the pupil location for >100 ms continuously were excluded from the analysis. Finally, we excluded all subjects for whom the combined rejection rate (from eye deviation and lost tracking) was >30% (7/37 subjects). Among the set of subjects who remained (*n* = 30), the median rejection rate was 8.6% [4.2–17.7%]. We also confirmed with a randomization test, based on the χ^2^-statistic (see below), that these rejected trials did not significantly alter the distribution of responses in the contingency table for any subject (*P* values for distributions before and after rejection: 0.99, median across subjects).

### Statistical Analysis

#### Contingency tables.

Subjects’ responses in the task were used to construct 5 × 5 stimulus-response contingency tables, with change locations relative to the cued location in the rows and response locations relative to the cued location in the columns; no change events and responses were represented in *row 5* and *column 5*, respectively. Thus each contingency table comprised five categories of responses: hits (H), misses (M), false alarms (FA), mislocalizations (ML), and correct rejections (CR; Fig.1*F*). Since six values of orientation changes were tested at each location, the contingency table contained 100 independent observations: 24 (6 × 4) hits, 72 (6 × 12) mislocalizations, and 4 false alarms (by definition, false alarms do not depend on orientation change magnitude). These correspond to the number of independent response proportions in the contingency table, and the m-ADC model was fit to these responses.

#### Psychometric and psychophysical functions.

**Fig. 2.**
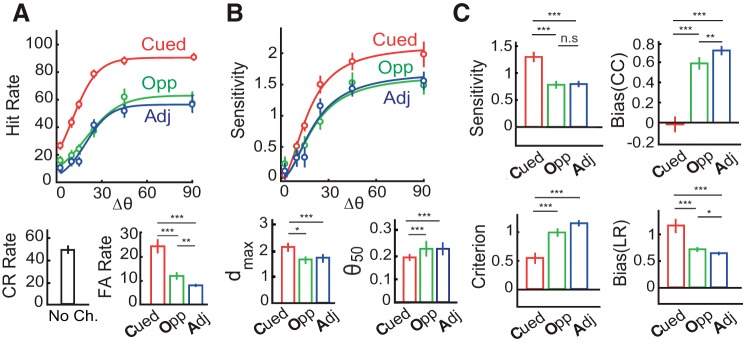
Sensitivity and bias changes induced by endogenous cueing. *A*, *top*: average psychometric function (*n* = 30 subjects) showing hit rates across different change-angles (Δθ). Curves: sigmoid fit. *A*, *bottom*: mean correct rejection (CR; *left*) and mean false alarm rates (FA; *right*). Error bars = jackknife SE. Color conventions are red: cued location (Cued); green: opposite to cue (Opp); blue: adjacent to cue (Adj), averaged across the ipsilateral and contralateral hemifields (*P < 0.05, **P < 0.01, ***P < 0.001). *B*, *top*: average psychophysical functions showing sensitivity at different change angles. Curves: Naka-Rushton fit. *B*, *bottom*: asymptotic sensitivity (d_max_; *left*) and change angle at half-maximum sensitivity (Δθ_50_; *right*). Other conventions are as in *A*. *C*: median sensitivity (*top left*), detection criterion (or detection threshold) (*bottom left*), choice criterion bias (b_CC_; *top right*), and likelihood ratio bias (b_LR_; *bottom right*) at different locations. Error bars = SE. Color conventions are as *A*.

To compute the psychometric function (percent correct as a function of orientation change angle), we calculated for each location, relative to the cue, the proportion of trials in which the subject detected and localized the change accurately, relative to all trials in which the change occurred at that particular location. Percent correct values, across all angles of orientation change, were fitted with a three-parameter sigmoid function to generate the psychometric function (Fig. 2*A*, *top*). Psychometric functions in 24/30 subjects were estimated with a set of six change angles spanning 2 to 90° (2, 10, 15, 25, 45, and 90°), presented in an interleaved, pseudorandomized order across locations. As the psychometric function tended to saturate at ~45°, the remaining 6/30 subjects’ psychometric functions were estimated with a set of 6 change angles spanning 2–45°. The analyses were repeated by excluding this latter set of six subjects, who were tested on the more limited range of angles, and in all cases we obtained results similar to those reported in the main text. A single, combined psychometric curve was generated by pooling contingencies across all subjects and computing the above metrics for the pooled data (Fig. 2*A*, *top*). False alarm and correct rejection rates were calculated based, respectively, on subjects’ incorrect and correct responses during no change trials.

#### Model fitting and prediction analyses.

To compute the psychophysical function (sensitivity as a function of orientation change angle), individual subjects’ response contingencies were fitted with the m-ADC model described previously. We estimated sensitivities and criteria with maximum likelihood estimation (MLE), using a procedure described previously (Sridharan et al. 2014). Sensitivity is expected to change depending on the magnitude of stimulus strength (change angle value); hence, different sensitivity values were estimated for each change angle tested. On the other hand, the criterion at each location was estimated as a single, uniform value across change angles; as change angle magnitudes at each location were distributed pseudorandomly, it is reasonable to expect that subjects could not anticipate and, hence, alter their criterion for different values of change angles. Thus, the model estimated 28 parameters (6 d′ values for each of the 4 locations and 4 criteria) from 100 independent observations in the contingency table.

We simplified m-ADC model estimation further using the following approach: as the probability of change was identical across the adjacent-ipsilateral and adjacent-contralateral locations (Fig. 1*E*, *left*), we compared the parameters (sensitivities, criteria) estimated for these two locations. Sensitivities and criteria were strongly correlated (opposite vs. adjacent: d′: ρ = 0.58, *P* = 0.002; c: ρ = 0.74, *P* = 0.0001) and not significantly different between these locations (difference of ipsilateral vs. contralateral medians: Δd′ = 0.07, Δc = 0.03, *P* > 0.5, Wilcoxon signed rank test). Thus, responses from cue ipsilateral and contralateral sides were averaged and treated as a single “adjacent” contingency in the contingency table. Therefore, the m-ADC model estimated 21 parameters (6 d′ values for each of the 3 locations, cued, opposite and adjacent, and their corresponding 3 criteria) from 63 independent observations in the contingency table. In all of the subsequent analyses, we report psychophysical parameters calculated for these three locations relative to the cue, treating the two cue-adjacent locations (A-I and A-C) as a single “adjacent” location (A). Occasionally, parameters are reported as a single value for uncued locations; these were based on their average values across all three uncued locations.

For the behavior prediction analysis (Fig. 1*H* and Supplemental Fig. S1, *B* and *C*) model fitting was done with only subsets of contingencies (hits and false alarms, hits and misses, or misses and false alarms), as described in results. The predicted contingency table was compared with the observed contingency table with a goodness-of-fit (randomization) test (see *Statistical tests, correlations, and goodness-of-fit*).

#### Psychophysical function fits.

The psychophysical function was generated by fitting the sensitivity values across angles at each location with a three-parameter Naka-Rushton function allowing asymptotic sensitivity (d_max_) and orientation change value at half-max (Δθ_50_) to vary as free parameters, keeping the slope parameter (n) fixed at 2; a value determined from pilot fits to the data (Herrmann et al. 2010). As before, a single combined psychophysical curve was generated by pooling contingencies across all subjects and computing the above metrics for the pooled data (Fig. 2*B*, *top*). Values of d_max_ and Δθ_50_ reported in the main text correspond to pooled psychophysical fits with jackknife error bars (Fig. 2*B*, *bottom left* and *right*). Tests of significant differences in d_max_ and Δθ_50_ values across locations were performed with a random permutation test: by comparing the true differences in values against a null distribution of differences calculated from contingency tables obtained by randomly shuffling, 1,000 times, the labels across change event locations (cued, opposite, and adjacent).

#### Computing psychophysical parameters and their modulations.

After fitting the m-ADC model to the contingency table to estimate sensitivity and criteria, we computed, at each location, the mean sensitivity (d′_av_; average value across change angles), as well as two measures of bias: the choice criterion (b_CC _= c-d′_av_/2), and the likelihood ratio (b_LR_; see Supplemental Data: Appendix, *Eq. 12*; also see results). For Gaussian decision variable distributions, both measures of bias are closely related (b_LR_ = exp[−d′ × b_CC_]).

In addition, we computed two measures to quantify attentional modulation of these parameters induced by endogenous cueing: a difference index and a modulation index. The attention difference index (ADI) for each parameter was computed as the difference between the values of the respective parameter at the cued location, and its average value across uncued locations (e.g., d′_ADI_ = d′_cued_ – d′_uncued_) whereas the attention modulation index (AMI) was computed as the ratio of the difference of the parameters at the cued and uncued locations to their sum [e.g., d′_AMI_ = (d′_cued_ – d′_uncued_)/(d′_cued_ + d′_uncued_)].

#### Statistical tests, correlations, and goodness-of-fit.

Unless otherwise stated, pairwise comparisons of different parameter values (e.g., mean d′ or bias) across locations were performed with the nonparametric Wilcoxon signed rank test with false discovery rate (Benjamini-Hochberg) correction for multiple comparisons. Correlations across different parameter values or modulation indices were typically Pearson correlations. For the optimal behavior, choice theory, and reaction time (RT) analyses, robust correlations (“percentage-bend” correlations) were computed to prevent outlier data points dominating the correlations (Wilcox 1994). Goodness-of-fit of the model to the data was assessed using a randomization test based on the χ^2^-statistic; the procedure is described in detail elsewhere (Sridharan et al. 2017). A small *P* value (e.g., *P* < 0.05) for the goodness-of-fit statistic indicates that the model fit deviated significantly from the observations.

To test whether there was covariation between sensitivity and bias within each subject’s data, we adopted the following procedure: two contingency tables were constructed for each subject, with responses drawn from the first half of her/his respective session (first one-half of the trials) and the second half (last one-half of trials). Psychophysical parameters were estimated from these two subsets of data yielding two measures of each psychophysical parameter per subject. An *n*-way ANOVA analysis was performed with each measure of bias (b_CC_, b_LR_) as the response variable and sensitivity as a continuous predictor, with subjects as random effects.

#### Model comparison analysis.

We compared the goodness of fit of our default model with two other models, one that assumed equal sensitivities at all uncued locations (m-ADC_eq-d_ model; d′_opp_ = d′_adj_) and one that assumed equal criteria at all uncued locations (m-ADC_eq-c_ model; c_opp_ = c_adj_). Models were compared with the Akaike Information Criterion (AIC), which represents a tradeoff between model complexity (the number of fitted model parameters) and goodness-of-fit (based on the log-likelihood function); a lower AIC score represents a better candidate model; similar results were obtained when using the Bayesian Information Criterion. The number of fitted parameters in the m-ADC_eq-d_ model was 15 (as compared with 21 for the standard m-ADC model) and was 20 in the m-ADC_eq-c_ model.

#### Analysis of RTs.

RTs were computed as the time from change onset to the time of response; no time limit was placed on responses, but subjects were asked to respond as quickly as possible. For each subject, trials in which RTs fell outside 3 standard deviations from their mean RT were considered outliers and excluded from further analysis. Each subject’s mean RT at each location was normalized by dividing by the median RT at the cued location across all subjects and correlated with psychophysical parameter estimates at the respective location. Partial correlations were performed to identify dependencies between RT and measures of bias while controlling for the effects of d′ and vice versa. We also performed multilinear regression analysis with RT as the response variable and change angle, mean sensitivity, and bias at each location as predictors; all predictors were scaled to zero mean and unit variance before the analysis. Regression coefficient magnitudes for sensitivity (β_d′_) and bias (β_b-LR_) were compared with a permutation test; we constructed a null distribution of regression coefficient differences by shuffling the location labels for the d′ and bias values randomly and independently across subjects. To discount the contribution of motor bias, “robust correlations” were computed between mean RTs and mean false alarm rates for each experimental block after subtracting the mean RT and false alarm values subject-wise to account for subject specific effects. An *n*-way ANOVA was applied, as before, treating RT as the response variable with false alarm rates as continuous predictors and subjects as random factors.

#### Analysis of optimal decisions.

Optimal decisions, in the m-ADC model framework, seek to minimize risk or maximize utility (Supplemental Data: Appendix, *Eq. 3*). Under certain assumptions (e.g., the costs associated with all types of errors are identical), optimal decision surfaces comprise hyperplanes in the multidimensional decision space. Our model fitting analysis suggested that observers’ choices were consistent with having employed this family of planar decision surfaces (as defined in Supplemental Data: Appendix, *Eqs. 12*, *13*, and *14*). But how did subjects decide which specific planar decision surface to adopt from among this family?

One possibility is that subjects sought to maximize the number of correct responses and minimize the number of errors. In this case, cost ratio at location j, β_obs-j_ = (C^0^_0_ − C^0^_j_)/(C^j^_j_ − C^j^_0_) = (C_CR_ − C_FA_)/(C_Hit_ − C_Miss_) = 1 (where C*^x^_y_* is the cost of responding to location *y* when the event occurred at location *x*). Yet, we noticed that β_j_, calculated as β_j_= (p_j_/ p_i_)/b_LR-j_ (Supplemental Data: Appendix, *Eqs. 8*, *10*, and *11*) systematically deviated from 1 across the population of subjects (Fig. 4*F*), suggesting that subjects did not assume an equal relative cost for false alarms versus correct rejections and misses versus hits. We also noticed that β_obs_ was different for the different locations, being predominantly greater than 1 at the cued location and less than 1 at uncued locations (Fig. 4*F*). Since there is only one type of correct rejection response, and since the model assumes that the cost for false alarms to each location is identical (Supplemental Data: Appendix, *Eq. 7*), it would be implausible to assume that subjects adopted a different relative cost of hits to misses at the different locations (cued, opposite, adjacent). Rather, we proposed that the reason for the difference in β_obs_ across the different locations was because each subject assumed a single, common cost ratio (β^s^_opt_) at all locations but deviated from this (subjectively) optimal ratio at some locations where she/he did not consider it necessary to perform optimally. To determine this subject-specific β^s^_opt_, we tested different values of β and selected the one that minimized the deviation of the actual risk (Risk^obs^ = Σ_i_Σ_j_C^i^_j_p_obs_^j^_i_) from the optimal Bayes risk (Risk^opt^ = Σ_i_Σ_j_C^i^_j_p_opt_^j^_i_) assuming a single β^s^_opt_ at all locations (ΔRisk^obs-opt^ = Σ_i_Σ_j_C^i^_j_[p_obs_^j^_i_ – p_opt_^j^_i_]); again, this deviation is zero if the subject did not deviate from β^s^_opt_ at any location. After some algebra, it can be shown that C^i^_j_ for hits and misses (or mislocalizations) can be written as a function of β^s^_opt_, C_FA_, and C_CR_ (see Supplemental Data: Appendix, *Eqs. 6*, *8*, and *9*, along with the following assumptions: *1*) C_Hit_ = C_CR_/β^s^_opt_; *2*) C_Miss_ = C_FA_/β^s^_opt_ ; and *3*) C^j^_i_ = C^j^_0_ for all i ≠ j, j ≠ 0), whereas p_obs_^j^_i_ can be written as a function of β_j_ and d_i_, and p_opt_^j^_i_, as a function of β^s^_opt_ and d_i_.

Based on this inferred optimal cost ratio (β^s^_opt_), three indices of optimal performance were computed for each subject. Two indices quantified the optimality of overall performance: *1*) an objective suboptimality index (SI_O_) defined as the deviation of the subject’s cost ratio from an objectively optimal cost ratio (β_opt _= 1) for maximizing correct responses, and computed as the magnitude of the logarithm of the ratio of β^s^_opt_ to β_opt_ (SI_O _= |log(β^s^_opt_ /β_opt_)|); and *2*) a global suboptimality index (SI_G_), defined and computed as the magnitude of the deviation of the optimal Bayes risk from the actual risk (SI_G_ = ΔRisk^obs-opt^). A third, locational suboptimality index was defined as the deviation of the cost ratio at each location from the subject’s own optimal cost ratio and computed for each location (SI_L_) as the magnitude of the logarithm of the ratio of β_obs_ (observed cost ratio) at that location and the subject’s own β^s^_opt_ (SI_L_ = |log (β_obs_/β^s^_opt_)|). These suboptimality indices were correlated with sensitivities, biases, and their modulations using “robust correlations.”

#### Risk curvature as a measure of bias.

We sought to develop a measure of bias that was different from the conventional SDT bias measure (Supplemental Data: Appendix, *Eq. 12*). We developed a risk curvature measure of bias, based on the following reasoning. It is reasonable to assume that subjects would afford greater attentional bias to a location if greater penalties for incorrect (or suboptimal) decisions occurred at that location, as compared with other locations. We propose that the differential curvature of the risk (or utility) function (∆R_C_) is a measure of this attentional bias. The risk measures the total average cost associated with a specific decision strategy, given sensory evidence, priors, and payoffs (Supplemental Data: Appendix, *Eqs. 11-12*). The curvature of this risk (R_C_) measures the “sharpness” with which the risk increases on either side of the optimum criterion. Briefly, a higher curvature of the risk function at a location entails a greater differential penalty (increase in risk) for suboptimal placement of the choice criterion at that location, Hence, subjects are expected to place their criterion with greater precision at locations of higher risk curvature (e.g., Fig. 5*A*). We employed the curvature (second derivative) rather than the slope (first derivative) for measuring this bias, because the slope of the risk curve at the optimal criterion is zero at all locations. In the probabilistically cued attention task, the cued location is more important for decisions than uncued locations. Consequently, a higher value of risk curvature should occur at the cued location (Fig. 5*B*, red; simulated data, see below) as compared with other, uncued (irrelevant) locations (Fig. 5*B*, blue). The difference between the magnitude of the risk curvatures at the cued and uncued locations (differential risk curvature or ∆R_C_) was used to quantify attentional bias toward the cued location.

#### Risk curvature analysis for simulated attention task.

We simulated an m-ADC task with two potential stimulus locations (Fig. 5, *A*–*D*). We denote the signal probability at cued and uncued locations as p_s-cued_ and p_s-uncued_ and the probability of no change as p_ϕ_. The cost ratio, β, was set to unity for both locations, corresponding to a goal of maximizing percent correct. To rule out the effects of sensitivity in these simulations, d′ at cued and uncued locations was set to identical values (d′_cued_ = d′_uncued_ = 1.0). To calculate risk curvature, we first determined the optimal criterion for both cued and uncued locations using the relationship between criteria, sensitivities, priors and payoffs for the m-ADC model (Supplemental Data: Appendix, *Eq. 11*). Using these sensitivity and criterion values, we generated the conditional response probabilities using m-ADC model equations. The risk (or utility) for each stimulus response contingency was calculated as the product of the cost and the probability of that contingency. The total risk (R) was then determined as the sum of the risks for all stimulus response-contingencies (Σ_i_Σ_j_C^i^_j_p_obs_^j^_i_) and across all locations. To determine risk curvature (R_C_), we varied the choice criterion about its optimum at each location across a range of values (±1.0), calculated the risk function at each value of the choice criterion, and then computed its second derivative. ΔR_C_ was calculated as the difference in R_C_ across cued and uncued locations.

#### Relationship between m-ADC choice criteria and alternative measures of spatial attention bias.

To test for correlation between m-ADC choice criteria and differential risk curvature (∆R_C_) in our data, first we estimated sensitivity, criteria, and ∆R_C_ for individual participants, using the same procedure as described above. Because our task lacked an explicit payoff structure, for these analyses we calculated the risk based on the observer-specific subjective cost ratio (β^s^_opt_), as reported in the decision optimality analyses (assuming a uniform cost ratio of β = 1.0 produced similar results).

We tested whether ∆R_C_ was correlated with cue-induced modulation of bias (both CC and LR) and d′, using robust correlations (percentage-bend). To test whether these relationships would be better fit with a quadratic function (second degree polynomial) relative to the linear fit, we determined normalized sum of squared residuals for each fit, calculated as ‘sse’/(*n*-*m*-1), where ‘sse’ is the sum of squared residual of fit; *n* is the number of samples or data points and *m* is the order of the polynomial (a lower value represents a better fit of the model to the data). Quadratic and linear fits were also compared using adjusted *R*^2^ (coefficient of determination) values, which measure how well the predictors explain the data while controlling for the number of predictors. A higher adjusted *R*^2^ is indicative of more informative predictors. We further performed a multiple linear regression analysis, with ∆R_C_ as the response variable and ADI-bias (b_LR_) and ADI-d′ as independent factors. To determine which of these factors exhibited a stronger relationship with ∆R_C_, we computed incremental *R*^2^ values associated with each factor by calculating the increase in adjusted *R*^2^ upon adding each factor into a multiple regression model. A larger value of incremental *R*^2^ for a factor indicates a stronger linear relationship with the response variable.

Finally, we computed another measure of attentional bias that did not involve computing the risk curvature. For each participant and each location, we calculated σ(b_CC_): the standard deviation of the choice criterion (b_CC_) values estimated with a jackknife approach. We correlated the AMI index of σ(b_CC_) against the AMI indices of d′ and bias (b_LR_) with robust correlations.

## RESULTS

### A Model for Predicting and Fitting Behavior in Multialternative Attention Tasks.

We developed a psychophysical (SDT) model for analyzing behavioral responses in endogenous cueing tasks of the type shown in Fig. 1*A*. In this task, the observer is cued to attend to one of two (or multiple) stimulus locations. At a random time following cue onset, an event of interest, for example, a change in grating orientation, occurs at one of the locations (“change” trials). On some trials, no events (changes) occur at any location (“catch” trials). The observer must detect and report the location of the change or indicate that no change occurred. Such tasks typically employ probabilistic spatial cues, the predictive validity of which varies across locations, and do not employ post hoc response probes. And, unlike response probe tasks (e.g., Rahnev et al. 2011; Wyart et al. 2012), such tasks enable measuring a spatial choice bias across locations. This is because, on each trial, the subject must make a single choice by comparing sensory evidence across multiple locations to detect and localize the change.

We measured the effect of endogenous cueing on sensitivity and bias by extending the recently developed m-ADC model framework (Sridharan et al. 2014). We extended the model for multialternative attention tasks that employ the method of constant stimuli, i.e., tasks in which stimuli can occur at different, unpredictable strengths at each location (Fig. 1*C* and Supplemental Fig. S1*D*). This new model is relevant for studies that seek to measure the effect of attention on the psychometric function at cued and uncued locations (Sridharan et al. 2017). The decision surface in this model comprises a family of intersecting hyperplanes in a multidimensional decision space (Fig. 1*C*). Parameterized by criteria, one at each location, these hyperplanes belong to a family of optimal decision boundaries for distinguishing each class of signal from noise (e.g., change at a given location versus no change) and provide a close approximation to optimal decision boundaries for distinguishing signals of one class from another (e.g., changes at one location from another; Supplemental Fig. S1, *D* and *E*). The model can be used to quantify perceptual sensitivities and choice criteria from stimulus-response contingency tables for tasks with any number of alternatives. An intuitive explanation of the model is presented in *m-ADC Model Description*, and further details can be found in Sridharan et al. (2014, 2017).

First, we fit a 4-ADC model for each subject’s 5 × 5 stimulus-response contingency table obtained from the five-alternative task (exemplar table in Fig. 1*F*). For these analyses, responses across two of the uncued locations (ipsilateral and contralateral) were averaged into a single contingency (adjacent) because responses were not significantly different across these locations (*P* > 0.2 for hits, misses, and false alarms at these locations, signed rank test; also see materials and methods and Supplemental Fig. S2*A*). This simplification significantly reduced the number of model parameters to be estimated (see materials and methods). Goodness-of-fit *P* values obtained from a randomization test (based on the χ^2^ statistic) were generally >0.7 (median: 0.84; range: 0.57–0.98; Fig. 1*G*, *bottom inset*), indicating that model fits did not deviate significantly from observers’ response proportions in the contingency table for this multialternative task.

To validate the model further, we tested the model’s ability to predict individual subjects’ responses. For this we fit the model using only a subset of the observers’ behavioral choices (33–58%) and tested its ability to predict their remaining (42–67%) choices (Fig. 1*H* and Supplemental Fig. S1,* B* and *C*). Three different subsets of contingencies were selected for fitting: *1*) hits and false alarms (Fig. 1*H*), *2*) false alarms and misses (Supplemental Fig. S1*B*), or *3*) hits and misses (Supplemental Fig. S1*C*). Correct rejection responses were included either implicitly (*cases 1* and *2*) or explicitly (*case 3*; see materials and methods for details). The model was able to predict all of the remaining observations in the contingency table with high accuracy (Fig. 1*H*; Supplemental Fig. S1, *B* and *C*; *P* values of randomization fit for predictions (median [95% confidence interval]): *case 1*: 0.69 [0.58–0.87]; *case 2*: 0.33 [0.25–0.48]; and *case 3*: 0.60 [0.50–0.75]).

Taken together, these results demonstrate that the 4-ADC model accurately fit observers’ behavioral responses in this five-alternative attention task.

### Endogenous Cueing Effects on Sensitivity, Choice Criterion, and Bias

In this task, did endogenous cueing of attention produce changes of sensitivity, changes of bias, or both? To answer this question, we varied the probability of change across the cued and uncued locations: changes were twice as likely at the cued location (66.7%), as all of the other locations combined (opposite: 16.7%, adjacent ipsi/contralateral: 8.3% each, Fig. 1*E*, *left*). We tested the effect of these different cue validities on modulations of sensitivity and bias, as estimated with the m-ADC model.

We clarify a few key terms used in the subsequent description of the results. First, we note the distinction between the terms “criterion” and “choice criterion” (or decision criterion). “Criterion,” here, refers to the “detection threshold,” whose value is measured relative to the mean of the noise distribution (e.g., Fig. 1*C*). Choice criterion (or b_CC_), on the other hand, is a measure of bias that was quantified as the deviation of the subject’s criterion (detection threshold) from the midpoint of the means of the signal and noise distributions (Macmillan and Creelman 2005). The lower the choice criterion at a location, the lower the value for the decision variable at which the subject chooses to indicate signal over noise at that location and, consequently, the higher the choice bias (for signal events) at that location. On the other hand, the likelihood ratio measure of bias (or b_LR_) was calculated as the ratio of the conditional probability density of the decision variable, at the value of the choice criterion, on change versus no change trials (Supplemental Data: Appendix). The higher the likelihood ratio bias at a location, the higher the choice bias (for signal events) at that location. Both choice criterion (b_CC_) and likelihood ratio (b_LR_) measures were used to quantify bias in this study.

Before the analysis of psychophysical parameters, we tested whether subjects were utilizing information provided by the cue regarding the location of the imminent change. We quantified the effect of cueing on “raw” performance metrics (psychometrics), including hit, false alarm, and correct rejection rates (Fig. 2*A*). Subjects (*n* = 30) exhibited highest hit rates for changes at the cued location as compared with the other locations, across the range of angles tested (Fig. 2*A*, *top*; *P* < 0.001 for difference in hit rates between cued vs. opposite and cued vs. adjacent; Wilcoxon signed rank test, Benjamini-Hochberg correction for multiple comparisons), indicating that subjects were indeed heavily relying on the cue to perform the task. Nevertheless, false alarm rates were also highest at the cued location (Fig. 2*A*, *bottom right*; cued vs. opposite: *P* = 0.0014; cued vs. adjacent: *P* < 0.001). We asked whether this pattern of higher hit rates concomitantly with higher false alarm rates occurred due to a higher sensitivity at the cued location, a higher bias (lower choice criterion) at the cued location, or both (see materials and methods).

Sensitivity was consistently highest at the cued location as compared with the other, uncued locations (*P* < 0.001; Fig. 2*B*, *top*). We fit the 4-ADC model incorporating a parametric form of the psychophysical function (the Naka-Rushton function). Asymptotic sensitivity (d_max_) was highest for the cued location as compared with each of the uncued locations (*P* < 0.001, permutation test against a null distribution of differences generated by randomly shuffling location labels; see materials and methods; Fig. 2*B*, *bottom left*) whereas change magnitude corresponding to half-maximum sensitivity (Δθ_50_) was significantly lower for the cued, as compared with each of the uncued, locations (*P* < 0.001; Fig. 2*B*, *bottom right*). Similarly, bias was uniformly highest at the cued location compared with the uncued locations: choice criteria were lowest, and likelihood ratio bias highest, toward the cued location compared with the other locations (Fig. 2*C*; *P* < 0.001; corrected for multiple comparisons).

Next, we asked whether these modulations of sensitivity and bias reflected a benefit (relative to baseline) at the cued location, a cost at the uncued locations, or both. For this, a subset of the subjects (*n* = 10) who were tested on the cued detection task were also tested on a neutrally cued version of the task presented in interleaved blocks (see materials and methods). In this task, changes were equally likely (25%) at each of the four locations. Within this pool of subjects, sensitivity for the neutrally cued locations was significantly lower than that at the cued location (*P* = 0.004) but only marginally significantly different from that at the uncued locations (*P* = 0.049; Supplemental Fig. S2*D*, *top*). On the other hand, biases (b_CC_ and b_LR_) for the neutrally cued locations were significantly different from cued (*P* = 0.002) as well as uncued (*P* < 0.01) locations (Supplemental Fig. S2*D*, *bottom*).

In sum, these results show that endogenous cueing of attention produced both a higher sensitivity as well as higher bias toward the cued location relative to uncued locations. The increase in sensitivity manifested primarily as a benefit at the cued location. In contrast, cueing produced both a strong benefit in bias at the cued location and a strong cost at uncued locations, relative to baseline.

### Sensitivity and Bias: Same or Different Mechanisms?

**Fig. 3.**
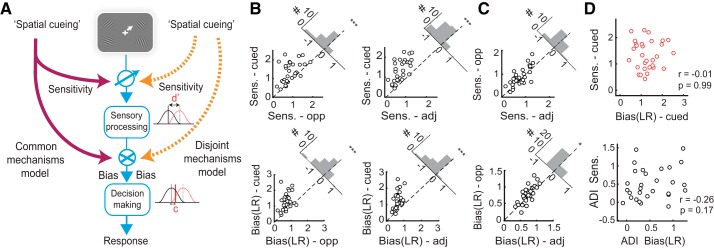
Cueing-induced changes in sensitivity and bias are dissociable. *A*: schematic of models by which spatial cueing may modulate sensory processing (sensitivity, d′) and decision-making (bias or criterion). Cueing may modulate sensitivity and bias through a common mechanism (purple arrows) or through distinct mechanisms (orange arrows). *B*: sensitivity (*top*) or bias (*bottom*) at the cued versus opposite (*left*) or adjacent (*right*) locations, measured with the 4-signal detection (4-ADC) model. Data points: individual subjects (*n* = 30). Dashed diagonal line: line of equality. *Top right insets*: histograms indicating difference in parameter values. *P < 0.05, ***P < 0.001, for a difference of medians. *C*: same as in *B* but for the opposite versus adjacent locations. Other conventions are as in *B*. *D*, *top*: covariation between sensitivity and bias at the cued location. *D*, *bottom*: covariation between difference indices (ADI) of sensitivity and bias. LR, likelihood ratio. Data points: individual subjects.

Are the enhancements of sensitivity and bias with endogenous cueing mediated by the same mechanism or different mechanisms? To answer this question, we evaluated two competing models (Fig. 3*A*). According to the “common” mechanism model, cue-induced selection biases compete for sensory processing resources to enhance sensitivity, so that sensitivity at each location covaries systematically with bias at that location. On the other hand, according to the “disjoint” mechanisms model bias and sensitivity modulation are decoupled and independent of each other. We sought to distinguish between these two models by examining several lines of evidence.

First, cue validity at the uncued locations occurred at one of two different values (Fig. 1*E*, *left*). Of the one-third of change trials occurring at the uncued locations, changes were twice as likely at the cue-opposite location (16.7%) as at either of the adjacent locations (8.3% each). We asked whether these different cue validities would produce systematically differing values of sensitivity and bias at the different uncued locations. Across the population of subjects (*n* = 30), sensitivities at the opposite location were not significantly different from those at the adjacent locations (*P* = 0.08; Fig. 2*C*, *top left*, and Fig. 3, *B* and *C*, *top*). In contrast, choice criterion bias (b_CC_) was significantly lower for the opposite as compared to the adjacent locations (*P* = 0.009; Fig. 2*C*, *top right*), and likelihood ratio bias (b_LR_) was significantly higher for the opposite as compared to the adjacent locations (*P* = 0.018; corrected for multiple comparisons). Taken together with the graded variation in bias in the neutrally cued condition (Fig. 2*C*, *bottom right*, and Fig. 3, *B* and *C*, *bottom*, and Supplemental Fig. S2*D*), these results indicate that bias, rather than sensitivity, systematically modulated with endogenous cue validity.

We further tested this by evaluating the performance of two modified m-ADC models, one with sensitivity constrained to be equal across uncued (opposite and adjacent) locations (m-ADC_eq-d_) and one with criteria similarly constrained (m-ADC_eq-c_). Model selection analysis based on the AIC revealed that the m-ADC_eq-d_ model significantly outperformed both the m-ADC_eq-c_ model and the standard m-ADC model (AIC: m-ADC_eq-d_ = 500.1 [466.2–602.9]; m-ADC_eq-c_ = 508.6 [475.5–614.2]; and m-ADC = 508.7 [474.4–614.3]; *P* < 0.001, Wilcoxon signed rank test), whereas there was no significant difference in the performance of the m-ADC_eq-c_ and the standard m-ADC models (*P* = 0.12). The significantly higher evidence in favor of a model that incorporated identical sensitivities, but distinct criteria, at all uncued locations confirms that criteria, rather than sensitivities, varied in a graded manner with endogenous cue validity.

Next, we asked whether subjects who exhibited higher sensitivity at the cued location also exhibited higher bias at the cued location. Sensitivity and criteria (detection thresholds) were strongly positively correlated at the cued location (ρ = 0.6, *P* < 0.001) indicating that across observers, criteria covaried with sensitivities, in line with a key prediction of the m-ADC model for optimal decisions in this task (Supplemental Data: Appendix, *Eq. 11*). On the other hand, neither choice criterion nor likelihood ratio bias were correlated with sensitivity (ρ_CC-d′_ = 0.13, p_CC-d′_ = 0.49; ρ_LR-d′_ = 0.01, p_LR-d′_ = 0.99; Fig. 3*D*, *top*). The uncued locations (opposite, adjacent) revealed a similar trend of a positive correlation between sensitivity and criteria and no correlation (b_CC_) or even a negative correlation (b_LR_) between sensitivity and bias (Supplemental Fig. S3*A*). These results demonstrate that although endogenous cueing produced both the highest sensitivity and bias at the cued location, there was no evidence of a positive correlation between these quantities.

Although sensitivity and bias at the cued location did not covary, we asked whether subjects who showed the greatest differential sensitivity at the cued location (relative to uncued locations) also showed the greatest differential bias toward the cued location. To answer this question, we tested whether sensitivity and bias modulations, measured either as a difference index (ADI) or a modulation index (AMI) across cued and uncued locations (see materials and methods), were correlated. Neither bias value (choice criterion or likelihood ratio) significantly covaried with sensitivity, as measured with the difference index (ρ_CC-d′ _= −0.10, p_CC_ = 0.61; ρ_LR-d′ _= −0.26, p_LR_ = 0.17; Fig. 3*D*, *bottom*, and Supplemental Fig. S3*B*), or with the modulation index (ρ_CC-d′ _= −0.25, p_CC_ = 0.19; ρ_LR-d′_ = 0.25, p_LR_ = 0.18). Next, we tested whether sensitivity and bias were comodulated across experimental blocks in individual subjects. We divided data from each subject’s experimental session into two subsets of contiguous experimental blocks. Difference indices (ADI) or modulation indices (AMI) for bias from each block were subjected to an *n*-way ANOVA with the corresponding sensitivity (ADI or AMI) index as a continuous predictor and subjects as random effects. This analysis revealed that neither measure of bias significantly covaried with sensitivity, as measured by either modulation index (ADI or AMI), across blocks (*P* > 0.1, for all tests).

Finally, we examined the possibility that the graded variation of bias with cue validity was a consequence of parametric assumptions in the m-ADC model by comparing with a “similarity choice model” (Luce 2005). In this model, sensitivities and biases are estimated, not from a parametric, latent variable model, but by a factoring of the underlying probability densities. Sensitivity and bias parameters estimated by the similarity choice model were strongly correlated with the corresponding m-ADC model parameters (ρ = 0.7–0.9, *P* < 0.001) and also exhibited identical trends, including a graded variation in bias with cue validity and the lack of correlation between sensitivity and bias or their modulations.

To summarize, sensitivity enhancements were strongest at the cued location and not significantly different across uncued locations. Bias modulations were graded across locations and varied systematically with endogenous cue validity. Moreover, modulations of sensitivity and bias by endogenous cueing were uncorrelated within and across subjects. These results suggest that the “spotlight” model of attention applies primarily to attention’s effects on sensitivity, rather than bias. Moreover, the results overwhelmingly favor the hypothesis that sensitivity and bias changes are mediated by dissociable mechanisms (Fig. 3*A*, *right*) in this predictively cued attention task.

### Covariation of Sensitivity and Bias with RTs and Decision Optimality Indexes

Attention produces systematic effects on perceptual decisions, both in terms of faster RTs (Eason et al. 1969) and by influencing optimality of decision-making (Eckstein et al. 2013). To further disambiguate the “common” from the “disjoint” mechanism models, we tested whether subjects' RTs and decision optimality metrics covaried with sensitivity or bias. The results are reported here for likelihood ratio measure of bias (b_LR_); similar trends were observed for choice criteria (b_CC_)_._

**Fig. 4.**
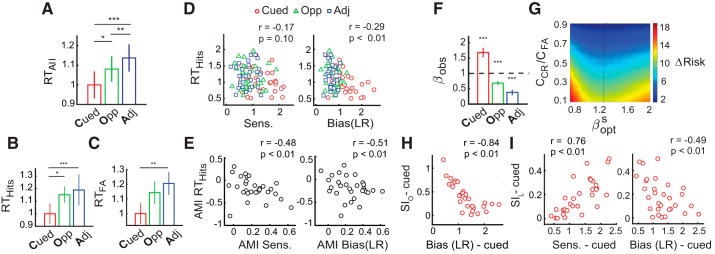
Reaction times (RTs) and decision optimality indices covary selectively with bias. *A*: normalized median reaction times across subjects for each location (all trials). Color conventions are red: cued location (Cued); green: opposite to cue (Opp); blue: adjacent to cue (Adj), averaged across ipsilateral and contralateral hemifields. Error bars = SE (*P < 0.05, **P < 0.01, ***P < 0.001). *B*: same as in *A* but for hit trials. *C*: same as in *A* but for false alarm trials. *D*: covariation of normalized median reaction times on correct trials for each subject at each location with sensitivity (*left*) or bias (*right*). Data points: values for individual subjects at particular locations. Color conventions are as in *A*. LR, likelihood ratio. *E*: same as in *D* but covariation of the modulation indices (AMI) for median reaction times with those for sensitivity (*left*) or bias (*right*). Data points: individual subjects. Other conventions are as in *D*. *F*: median observed cost ratio (β_obs_) at the different locations. Dashed line: β_opt_ value (=1) for maximizing correct and minimizing incorrect responses. Color conventions are as in *A*. Error bars = SE. *G*: difference between the actual risk and the optimal Bayes risk (ΔR) for different values of β^s^_opt_ (*x*-axis) and different values of the cost ratio of false alarms to correct rejections (C_CR_/C_FA_; *y*-axis); data pooled across subjects. Warmer colors: larger values of ΔR. Black dots: β corresponding to minimal ΔR for each C_CR_/C_FA_. *H*: covariation of the objective suboptimality index (SI_O_) with bias at the cued location (see text for details). Data points: individual subjects. *I*: covariation of the locational suboptimality index (SI_L_) with sensitivity (*left*) and bias (*right*) at the cued location. Other conventions are as in *E*.

RT (normalized to cued location; materials and methods) for all (correct and incorrect) change responses varied in a graded fashion with cue validity: fastest RTs occurred for changes at the cued, followed by the opposite and adjacent, locations, in that order (*P* < 0.05, corrected for multiple comparisons; Fig. 4*A*). Similar graded trends were observed when the data were analyzed separately based on hit and false alarm responses (Fig. 4, *B* and *C*). This graded variation with cue validity suggested a close relationship between RT and bias.

**Fig. 5.**
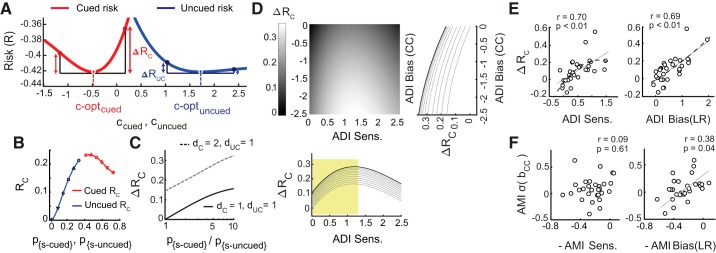
Differential risk curvature covaries selectively with bias. *A*: risk as a function of criteria at the cued (red curve) and uncued (blue curve) locations (simulation). Red and blue dots: optimal criteria (c_opt_) at the cued and uncued locations, respectively. Colored arrows: increase in risk for a fixed deviation (increase or decrease) of the criterion from its optimal value at each location. *B*: simulated variation of risk curvature (R_C_; second derivative of the risk function at the location of the optimal criterion) with signal probability (p_s_) at the cued (red) and uncued (blue) locations. Symbols on each curve: risk curvature values at each location, each pair corresponding to a different set of prior signal probabilities. *C*: simulated variation of differential risk curvature (∆R_C_; difference in risk curvature across the cued and uncued locations) with the ratio of signal probabilities at these locations. Solid line: d_cued_ = 1.00, d_uncued_ = 1.00; dashed line: d_cued_ = 2.00, d_uncued_ = 1.00. *D*: simulated variation of ∆R_C_ with attention difference index (ADI) bias (b_CC_) and ADI d′. Lighter shades: higher values of ∆R_C_. *Insets*: cross section showing variation of ∆R_C_ with each parameter, ADI b_CC_ (*right inset*) and ADI d′ (*bottom inset*), at fixed values of the other parameter. *E*: covariation of ∆R_C_, computed from behavioral data, with ADI d′ (*left*) and ADI bias (b_LR_; *right*). LR, likelihood ratio. Data points: individual subjects. Gray straight line: linear fit. Black dashed curve: quadratic fit. *F*: covariation of attention modulation index (AMI) for σ(b_CC_) with −AMI d′ (*left*) and −AMI bias (b_LR_; *right*), respectively. Other conventions are as in *E*.

Next, we quantified the relationship between RT, sensitivity and bias, considering only correct responses (hits). RT (normalized) and bias were robustly negatively correlated (Fig. 4*D*, *right*); ρ = −0.29, *P* = 0.006), whereas RT and d′ were not (Fig. 4*D*, *left*; ρ = −0.17, *P* = 0.10). To test whether the RT and bias correlation was rendered more robust by the latent covariation between RT and d′, we performed a partial correlation analysis of RT versus bias controlling for the effect of d′ (and vice versa). We found a significant negative partial correlation between RT and bias, (ρ_p _= −0.21, *P* = 0.04) whereas the partial correlation between RT and d′, controlling for bias, was not significant (ρ_p _= −0.19, *P* = 0.07). We then quantified the comodulation of RT with d′ and bias across cued and uncued locations. RT AMI was significantly negatively correlated with both bias AMI (ρ = −0.51, *P* = 0.004; Fig 5*E*, *right*), and d′ AMI (ρ = −0.48, *P* = 0.007; Fig. 4*E*, *left*). Nevertheless, multilinear regression analysis of RT (change angle, average d′, and bias as predictors; see materials and methods) revealed a more negative standardized regression coefficient for RT variation with bias as compared with d′, that approached significance (β_b-LR_ = −0.082 [−0.21 0.05], β_d′_ = −0.019 [−0.069 0.071] – median [95% confidence interval]; *P* = 0.046, bootstrap test for difference of β-magnitudes).

We also tested whether the pattern of RT correlations reflected a motoric response bias rather than a cue-induced choice bias. We correlated false alarm rates in each of the six experimental blocks with the mean RT on false alarm trials in that block, separately, for the cued and uncued locations. A significant correlation would indicate that faster responses (due to a motor bias) produced more false alarms and, correspondingly, a higher bias. Contrary to this hypothesis, we found no significant correlation between RT and false alarm rates across blocks at any location (cued: ρ = 0.05, *P* = 0.58; opposite: ρ = 0.13, *P* = 0.18; adjacent: ρ = −0.15, *P* = 0.10). These results were confirmed with an ANOVA with RT as the response variable and false alarms as continuous predictors, with subjects as random factors. Taken together, these results support a more robust covariation of RTs with bias changes, rather than with sensitivity changes, induced by spatial cueing.

Next, we tested for the covariation of sensitivity and bias with metrics of decision optimality. For this, we measured subjects’ observed cost ratio (β_obs_), defined as the ratio of the prior odds ratio to the bias at each location (Supplemental Data: Appendix, *Eq. 12*), and compared it with the optimal cost ratio (see materials and methods for details). In the m-ADC model decision rule framework (minimizing risk), β_opt_ equals the ratio of the cost of correct rejections versus false alarms to the cost of hits versus misses [β_opt_ = (C_CR_ − C_FA_)/(C_Hit_ − C_Miss_); Supplemental Data: Appendix, *Eq. 8*]. Therefore, subjects with a goal of maximizing successes and minimizing errors in our task should have assumed an optimal cost ratio of unity (β_opt _= 1) at all locations (Fig. 4*F*, dashed horizontal line). Yet, a vast majority of subjects exhibited systematic deviations from this optimum (Fig. 4*F*): the observed cost ratio was significantly greater than 1 at the cued location and significantly less than 1 at uncued locations (*P* < 0.001, signed rank test; Fig. 4*F*).

We investigated the reason for this systematic pattern of sub-optimalities. A first, potential scenario is that subjects’ observed cost ratio deviated from the optimal ratio because they perceived a signal probability (perceived prior) at each location that deviated systematically from the actual signal priors. This scenario arises when subjects fail to detect some proportion of changes, especially when the change in orientation was small. However, this explanation is not tenable because small orientation changes would be difficult to detect at both cued and uncued locations. Hence, the perceived prior ratio would have had to be lower than the actual ratio at all locations. While this scenario can account for the lower than optimal bias at the cued location, it cannot account for the higher than optimal bias at each of the uncued locations. An alternative scenario is that subjects assumed different cost ratios (β-s) at different locations, such that they judged errors arising from false alarms as costlier compared with errors arising from misses at the cued location (β > 1) and vice versa at uncued locations (β < 1). Nevertheless, no rational explanation can be readily conceived for subjects attributing different costs to false alarms and misses at the different locations. Having ruled out these alternative potential scenarios, we proposed the following hypothesis: each subject sought to minimize risk by adopting a “subjective” cost ratio (β^s^_opt_) that was uniform across all locations but differed from 1 (β^s^_opt_ ≠ 1) and was subjectively optimal to each observer's own estimate of the relative cost of false alarms and misses. The β^s^_opt_ for each observer was measured as the cost ratio (C_FA_/C_CR_; see materials and methods) that minimized the difference between the actual risk and the optimal Bayes risk (ΔR^obs-opt^). With data pooled across subjects, β^s^_opt_ occurred at a value of 1.3, across the range of C_FA_/C_CR_ values tested (Fig. 4*G*)

First, we examined two metrics, which measured the deviation from optimality of each observer’s overall performance (materials and methods): *1*) an objective suboptimality index (SI_O_), which measures the deviation of β^s^_opt_ from β_opt_; and *2*) a global suboptimality index (SI_G_), which measures the deviation of the observed risk from the optimal Bayes risk (ΔR^obs-opt^). SI_O_ was negatively correlated with bias at the cued location (ρ = −0.84, *P* < 0.001; Fig. 4*H*) but not at the uncued locations (*P* > 0.07), whereas it was not correlated with sensitivity at any location (*P* > 0.4). In contrast, SI_G_ was positively correlated with bias and negatively correlated with sensitivity at both opposite and adjacent locations (SI_G_ vs. bias: ρ_opp _= 0.71, *P*_opp_< 0.001; ρ_adj _= 0.81, *P*_adj _< 0.001; Supplemental Fig. S4*B*; SI_G_ vs. sensitivity: ρ_opp _= −0.61, *P*_opp _< 0.001; ρ_opp _= −0.39, *P*_opp _= 0.03) but not at the cued location (*P* = 0.153). Next, we quantified deviation from optimality at each location by defining a locational suboptimality index (SI_L_), which measures the deviation of the cost ratio at each location (β_obs_) from the individual’s optimal cost ratio (β^s^_opt_). SI_L_ was significantly lower (subjects more optimal) at the cued location than at either uncued location (*P* < 0.001; Supplemental Fig. S4*A*). SI_L_ was negatively correlated, across subjects, with bias at the cued location (ρ = −0.5, *P* = 0.006), whereas it was positively correlated with sensitivity (ρ = 0.76, *P* < 0.001; Fig. 4*I*).

In sum, subjects who exhibited a greater bias toward the cued location, and lower bias toward uncued locations, made more optimal decisions overall in this attention task (Fig. 4, *F*–*I*, and Supplemental Fig. S4, *A* and *B*). Decisional optimality was highest at the cued location and showed opposite patterns of correlations with sensitivity and bias (Fig. 4, *H* and *I*, and Supplemental Fig. S4, *A* and *B*). Taken together, this dissociation between sensitivity and bias in terms of RTs and decision optimality metrics further confirms the finding that sensitivity and bias effects were mediated by disjoint mechanisms in this predictively cued attention task.

### Distinguishing Mechanisms of Endogenous Attention from Expectation

Our results, thus far, suggest dissociable effects of endogenous spatial cueing on sensitivity and bias. While enhanced sensitivity toward the cued location is a commonly reported effect of spatial attention (Bashinski and Bacharach 1980; Ciaramitaro et al. 2001), do changes of choice criteria also reflect spatial attention’s effects (Summerfield and Egner 2009)? Recent studies have suggested that attention includes processes that selectively alter decisional policies (e.g., criteria) based on priors and payoffs (Luo and Maunsell 2018; Zénon and Krauzlis 2012). Changes of choice criteria induced by spatial probabilistic cueing, therefore, likely reflect a key component of attention (Buschman and Kastner 2015; Luo and Maunsell 2018). Nevertheless, we evaluated the possibility that differences in event expectation, arising from different prior probabilities of events (cue validities) at the cued versus uncued locations, modulated choice bias independently of attention in our task.

First, we tested whether trivial strategies, based on event expectation (prior probabilities) alone, biased subjects’ choice outcomes when no signal evidence was available. We measured false alarm rates to each location on “no change” trials (Fig. 1*F* and Supplemental Fig. S4*C*), which are indicative of each subject’s choice biases to the different locations in the absence of sensory evidence. We tested the distribution of these false alarm rates against three other distributions, each of which reflected alternative strategies by which choices could be influenced by priors alone. First, a decision strategy to maximize the proportion of correct responses, based on event expectation alone, which corresponds to consistently selecting the location with the greatest prior probability. Our data invalidated this hypothesis (*P* < 0.001, goodness-of-fit test for multinomial data, indicating mismatch of model to data; Supplemental Fig. S4*C*). Second, a probability matching strategy (Duda et al. 1980; Sugrue et al. 2005; Wozny et al. 2010) based on task specified priors, reflecting the relative proportion of change events at each location. In this case, the proportion of false alarms to each location should be distributed according to relative prior probabilities at each location. Our data also invalidated this hypothesis (*P* = 0.015; Supplemental Fig. S4*C*). Third, a probability matching strategy based on perceived prior probabilities, reflecting the relative proportion of detections at each location for each subject on change trials. Perceived priors were quantified as the proportion of choices to the cued and uncued locations on all change trials, combined (sum of *rows 1–4* of the contingency table; Supplemental Fig. S4*C*, unfilled bars with dashed outlines). We tested whether the proportion of false alarms to each location were distributed according to these perceived priors. Our data invalidated this hypothesis also (*P* = 0.0012; randomization test; Supplemental Fig. S4*C*). Overall, the inability of any of these distributions to describe false alarm rates in our data suggest that subjects’ choices could not be explained by event expectation based strategies alone.

Next, we developed an alternative measure of spatial attention bias, unrelated to event expectation, to validate our m-ADC bias: the differential curvature of the risk (or utility) function (detailed description in *Risk curvature as a measure of bias*). Briefly, a higher curvature of the risk function at a location entails a greater penalty for suboptimal placement of the criterion at that location, and vice versa (Fig. 5*A*). Therefore, subjects must place their choice criteria with greater precision (or care) at locations of higher risk curvature, to avoid significant penalties for suboptimal perceptual decisions at those locations.

We simulated a multialternative attention task (see materials and methods) to examine the relationship between risk curvature, signal probability and bias. We found that risk curvature was higher at the cued location as compared with the uncued location (Fig. 5*A*) in our multialternative task and varied systematically with the prior probabilities at each location (Fig. 5*B*). Differential risk curvature (∆R_C_), the difference of risk curvature between the cued and uncued locations, varied monotonically with the ratio of signal probabilities at cued and uncued locations (*P*_cued_/*P*_uncued_; Fig. 5*C*), despite identical sensitivities at these locations. These data indicate that differential risk curvature (∆R_C_) is a plausible measure of attentional bias: ∆R_C_ represents how much more precisely subjects must specify their choice criteria at the cued location versus at uncued locations. Finally, ∆R_C_ also varied monotonically with the difference of choice criteria across cued and uncued locations (ADI b_CC_; Fig. 5*D*, *right inset*). On the other hand, differential risk curvature varied nonmonotonically with the difference in d′ at cued and uncued locations (ADI d′; Fig. 5*D*, *bottom inset*).

We tested whether a relationship between m-ADC choice criteria and the ∆R_C_ measure of spatial attention bias was present in our data. First, we measured ∆R_C_ across cued and uncued locations in individual participants (see materials and methods) and tested whether it was correlated with cueing-induced modulation of bias (both b_CC_ and b_LR_) and d′. We observed that ∆R_C_ was correlated with both bias and d′ modulations (ADI; Fig. 5*E*). The latter is an expected result because the range of ∆d′ values in our data matched one (the rising) part of the nonmonotonic curve shown in Fig. 5*D*, *bottom inset* (shaded). Nevertheless, the data revealed evidence for a nonmonotonic relationship at larger values of ∆d′ (Fig. 5*D*, *bottom inset*). Consequently, we tested whether any of these relationships would be better fit with a nonlinear function (second degree polynomial) using the normalized sum of squared residuals as well as adjusted *R*^2^ (coefficient of determination) values. We observed that the normalized sum of squares residual measure was lower for a quadratic fit of ∆R_C_ vs. ∆d′ (SS_err_: quadratic = 13.8 × 10^−2^; linear = 14.5 × 10^−2^), whereas the measure was lower for a linear fit of ∆R_C_ vs. ∆b (b_CC_: quadratic = 16.0 × 10^−2^, linear = 15.4 × 10^−2^; b_LR_: quadratic = 11.2 × 10^−2^, linear = 10.8 × 10^−2^). Confirming this trend, the adjusted *R*^2^ was greater for a quadratic (vs. linear) fit for the variation with ∆d′ (adjacent *R*^2^: quadratic = 0.37, linear = 0.34), whereas the reverse was true for the variation with ∆b (b_CC_: quadratic = 0.27, linear = 0.30; b_LR_: quadratic = 0.49, linear = 0.51). These results indicate a monotonic variation of differential risk curvature with change in bias (both b_CC_ and b_LR_) but not with ∆d′ in this multialternative task. We also performed multiple linear regression with ∆R_C_ as a response variable and ∆b and ∆d′ as independent factors. We observed a higher standardized regression coefficient for ∆b as compared with ∆d′ (β: ∆b = 0.090, ∆d′ = 0.066), as well a higher incremental *R*^2^ (*R*^2^_inc_: ∆b = 0.035, ∆d′ = 0.018), further confirming a more robust, linear relationship of ∆R_C_ with m-ADC bias, rather than with d′ in this multialternative task.

Finally, we identified yet another measure of attention bias that did not involve computing the risk curvature. We hypothesized that high attention bias would produce lower variance of the choice criterion, possibly due to the significant penalty associated with suboptimal positioning of the choice criterion at cued locations (Fig. 5*A*). Hence, we estimated, for individual participants, the standard deviation of the choice criterion across blocks at each location, σ(b_CC_), and tested for its correlations with modulations of sensitivity and m-ADC bias. We observed that σ(b_CC_) modulations were significantly correlated with the modulations of bias (b_LR_; ρ = 0.381, *P* = 0.038; Fig. 5*F*, *right*) but not with sensitivity (ρ = 0.097, *P* = 0.611; Fig. 5*F*, *left*).

These results demonstrate that m-ADC model estimates of bias, but not sensitivity, varied systematically and monotonically with two independent measures of spatial attention bias, ∆R_C_ and σ(b_CC_). Taken together, these lines of evidence strongly support the hypothesis that cueing-induced changes of m-ADC bias in our attention task reflect spatial attention, rather than expectation, mechanisms. These observed trends, based on alternative measures of attention bias, ∆R_C_ and σ(b_CC_), further confirm our findings that, in this predictively cued attention task, sensitivity and bias changes were mediated by dissociable mechanisms.

## DISCUSSION

To understand the neural mechanisms by which attention operates in the brain, it is essential to tease apart its component processes. With a predictively cued, multialternative endogenous attention task (Posner et al. 1980), and a multidimensional signal detection model, we show that attention affects perceptual sensitivity and choice bias through dissociable mechanisms. Bias modulated systematically with endogenous cue validity whereas sensitivity enhancement was strongest at the cued location but not significantly different across uncued locations. These results suggest that in this attention task sensitivity control mechanisms allocated sensory processing resources in an all-or-none manner (“spotlight model”), whereas bias control mechanisms apportioned decisional weights in a more graded manner. Moreover, bias modulations were uncorrelated with sensitivity modulations, and bias, but not sensitivity, correlated strongly with key decisional metrics including reaction times and decision optimality indexes.

Among the earliest studies to employ attention tasks such as those employed in this study– in which the spatial cue indicates event probability— were those of Posner et al. (1980). Subsequently, these task designs were extensively used to study attention behaviors (Bashinski and Bacharach 1980; Cavanaugh and Wurtz; Cohen and Maunsell 2009; Corbetta et al. 2000; Müller and Findlay 1987; Steinmetz and Moore 2014; Williford and Maunsell 2006). Such a paradigm manipulates what Posner et al. (1980) termed “expectancy,” to align the “central attention system” with selected sensory input channels for making a perceptual decision. The key question in such tasks was whether the cost-benefit effects of attention occurred at a sensory stage, or a decisional/response stage through a modulation of criteria, and whether they were “independent, serial, or interactive.” In tasks with several alternatives, especially those with graded cue validity (such as our task), it remained to be shown whether performance improvements were mediated by shifts in criterion at different spatial locations, rather than by the action of a central attentional mechanism (Duncan 1980).

Do sensitivity and bias changes in our multialternative attention task, then, both reflect components of attention? There is emerging consensus that attention includes processes that selectively enhance sensory information processing (e.g., sensitivity) and also those that selectively alter decision policies for gating relevant information (e.g., choice criteria), based on sensory evidence, priors and/or payoffs (Buschman and Kastner 2015; Dosher and Lu 2000; Eckstein et al. 2009; Krauzlis et al. 2014; Luo and Maunsell 2018). By this definition, changes of choice criteria induced by spatial probabilistic cueing do reflect a key component of attention (Buschman 2015; Krauzlis et al. 2014; Luo and Maunsell 2015).

Nevertheless, the challenge of dissociating and quantifying criterion changes, as distinct from sensitivity changes, in multialternative attention tasks with spatial probability cues, was recognized in studies since the 1980s. For example, Duncan (1980) discussed this challenge for attention tasks requiring detection or discrimination among multiple alternatives. Such tasks entail comparing evidence across alternatives to make a decision. Correctly interpreting benefits or cost in performance for more or less probable stimulus alternatives would require differentiating between sensory capacity limitation effects and bias-induced criterion adjustments. In spite of this challenge being well recognized, an SDT framework for disentangling sensitivity from bias for such multialternative tasks was not available until recently (Sridharan et al. 2014, 2017). The m-ADC model, inspired by earlier SDT work (Ashby and Townsend 1986; Tanner 1956), models the multialternative decision in a multidimensional decision space and decouples sensitivity and criterion effects on behavioral performance (Sridharan et al. 2014). Applied to data from this study, the m-ADC model revealed that both bias changes and sensitivity changes independently contribute to performance benefits of endogenous attention.

Our study follows a rich literature of psychophysics studies that sought to distinguish endogenous attention’s effects on sensitivity and bias. For instance, Bashinski and Bacharach (1980) tested the effects of endogenous spatial cueing on sensitivity and bias, using a dot stimulus detection task with probabilistic cueing (80% valid cues). The study reported a benefit in sensitivity on the cued side, with little corresponding cost on the uncued side. Surprisingly, bias was found to be not different between cued and uncued locations. In contrast to these findings, Shaw (1984) reported that, in luminance detection tasks, focusing attention produced bias (criterion) changes without concomitant changes of sensitivity. Along similar lines, Müller and Findlay (1987) demonstrated results similar to those of Shaw (1984), showing that attention primarily produced changes of bias at the cued (relative to uncued) location in luminance detection tasks.

These contradictions can be readily explained by differences in the psychophysical models used to analyze these three-alternative task designs. Bashinski and Bacharach (1980) analyzed a three-alternative detection task with two one-dimensional models and grouped mislocalization responses with misses (Supplemental Fig. S1). They also partitioned the false alarm rates according to an ad hoc rule, a pitfall highlighted by other studies as well (Luck et al. 1996; Müller and Findlay 1987). Similarly, Müller and Findlay (1987) employed a two-stage signal detection model, which was unable to take into account all nine stimulus-response contingencies in their three-alternative task. These data were fit by ignoring miss and mislocalization responses, although these responses constituted a significant proportion of the overall responses. In general, analyses that ignore any category of response can produce inaccurate estimates of sensitivity and bias (Sridharan et al. 2014, 2017).

Similar analysis strategies were employed by recent studies that sought to identify neural correlates and mechanisms of sensitivity and bias (Chanes et al. 2013; Luo and Maunsell 2015). For example, Chanes et al. (2013) tested the roles of different frequencies of stimulation with rhythmic TMS over the right FEF, and reported frequency-specific effects of TMS on sensitivity and criterion. This study adopted a three-alternative task design in which subjects had to detect and report the location of a Gabor grating with one of three button presses (left, right, or neither). Again, because of the lack of an appropriate psychophysical model for analyzing this three-alternative task, mislocalizations were entirely removed from the analysis as “error” responses, an analysis strategy that could lead to incorrect estimates of sensitivity and bias.

Other previous studies (e.g., Luck et al. 1996) employed a post hoc response probe paradigm in which subjects were cued to attend to one of multiple locations, and target presentation was followed by a response probe. Subjects had to indicate whether or not a target had appeared at the response probe location, the cue being valid when the attentional cue matched the location of the response probe (response cue validity). Despite multiple potential stimulus locations, the response probe rendered this a 2-AFC (Yes/No) design that could be readily analyzed with independent one-dimensional signal detection models at each location. These studies have typically found systematic changes in sensitivity with response cue validity, but no reliable changes of bias (Luck et al. 1996; Wyart et al. 2012). In contrast, the m-ADC model revealed a graded variation of bias across cued and uncued locations, with bias being highest at the cued location (location of highest cue validity; Fig. 2*C*).

These differences may arise due to an essential distinction between 2-AFC response probe tasks and multialternative tasks with spatial probability cues, employed in this study (see Supplemental Data, Discussion: Distinction between response bias in 2-AFC tasks and choice bias in m-ADC tasks). Briefly, in response probe tasks, decisions need be based only on sensory evidence at the response probe location: there is no need to compare sensory evidence at the cued location against evidence at other, uncued locations. Therefore, criteria in such response probe tasks likely reflect event expectation, rather than spatial attention mechanisms (Summerfield and Egner 2009; Wyart et al. 2012, but see Rahnev et al. 2011). In contrast, for multialternative attention tasks such as the one employed in this, and previous studies (e.g., Cohen and Maunsell 2009; Müller and Findlay 1987; Wang and Krauzlis 2018), signal probability is the only relevant attention cue. Unlike response probe tasks in which independent Yes/No decisions are made at each location, such multialternative attention tasks require subjects to detect and localize the stimulus (change) event by directly comparing sensory evidence across cued and uncued locations, on every trial. The modulation of m-ADC bias (difference in b_CC_ or b_LR_ across cued and uncued locations) by spatial cueing, therefore, reflects a decision policy that affords greater weight to sensory evidence at the cued versus uncued locations. Consequently, choice criteria, as measured with the m-ADC model, represent a measure of attention bias.

To quantify this relationship further, we formulated a different measure of spatial attention bias, the differential risk curvature or ∆R_C_, which measures how sharply the risk increases when making suboptimal decisions at the cued versus at the uncued locations. We propose that processes linked to attention are likely to control the precision with which the subject specifies her/his choice criteria at cued versus uncued locations. Due to differences in risk profiles (R_C_) at each location (Fig. 5*A*), subjects would place their criteria with more precision (less variance) at the cued location, compared with the uncued location. Moreover, ∆R_C_ is unlikely to be related to signal expectation: while signal expectation arises from stimulus statistics (e.g., event probability), it is unlikely to be associated with the evaluation of differential risks (or benefits) at the cued and uncued locations. We discovered that ∆R_C_ correlated strongly with m-ADC bias, but not sensitivity, both in simulations and in real data. Finally, m-ADC choice bias, but not sensitivity, also correlated strongly with an independent, data-driven measure of attentional bias: σ(b_CC_), which quantifies the standard deviation of the choice criterion across blocks (Fig. 5*F*). These converging lines of evidence further confirm that m-ADC choice criteria represent a measure of spatial attention, and not signal expectation, bias.

Previous studies have reported diverse effects of attention on neural firing, tuning functions, noise correlations, and synchrony (Martinez-Trujillo and Treue 2004; McAdams and Maunsell 1999; Reynolds et al. 2000; Spitzer et al. 1988; Zénon and Krauzlis 2012). A possible explanation for these diverse reports is that endogenous cueing of attention engages multiple processes, each with a distinct behavioral consequence and neural signature. The m-ADC model opens up exciting new avenues for identifying the precise component of attention that could underlie these effects in diverse, multialternative attention paradigms.

## GRANTS

This research was funded by a Wellcome Trust/Department of Biotechnology India Alliance Fellowship [IA/I/15/2/502089], a Science and Engineering Research Board Early Career award [ECR/2016/000403], a Pratiksha Trust Young Investigator award, a Department of Biotechnology-Indian Institute of Science Partnership Program grant, a Sonata Software grant, and a Tata Trusts grant (to D. Sridharan).

## DISCLOSURES

No conflicts of interest, financial or otherwise, are declared by the authors.

## AUTHOR CONTRIBUTIONS

D.S. conceived and designed research; S.B. and S. Grover performed experiments; S.B., S. Grover, and S. Ganesh analyzed data; S.B., S. Grover, S. Ganesh, and D.S. interpreted results of experiments; S.B. and S. Ganesh prepared figures; D.S. drafted manuscript; D.S. and S.B. edited and revised manuscript; D.S. approved final version of manuscript.

## ENDNOTE

Supplemental figures and data, along with datasets generated and analyzed during the current study, are available on: https://figshare.com/s/b4b1f34ae4087420bd85.
